# Non-ischemic Cardiomyopathy Secondary to Left Ventricular Hypertrophy due to Long-term Anabolic-androgenic Steroid Use in a Former Olympic Athlete

**DOI:** 10.7759/cureus.3313

**Published:** 2018-09-17

**Authors:** Edward T Ha, Michael L Weinrauch, Jeffrey Brensilver

**Affiliations:** 1 Medicine, St. George's University, West Indies, GRD; 2 Cardiology, Overlook Medical Center, Summit, USA; 3 Internal Medicine, Overlook Medical Center, Summit, USA

**Keywords:** anabolic steroids, cardiomyopathy, arrhythmias, heart failure, ventricular tachycardia, left ventricular hypertrophy, androgenic steroids, non-ischemic cardiomyopathy

## Abstract

Currently, the cardiovascular risk associated with the use of anabolic steroids is not well documented. Recent studies have shown that its use may potentiate the development of cardiac dysfunction in the short term. This case report describes an encounter that supports a causal link between anabolic-androgenic steroid use (AAS) and cardiomyopathy later in life. We herein present a case study of a 73-year-old prior Olympic athlete who had misused AAS for 20 years and subsequently was found to have developed a systolic and diastolic cardiomyopathy, presumably due to long-standing left ventricular hypertrophy. A 73-year-old man presented to our medical center with symptoms of lightheadedness and palpitations. He was found to be in ventricular tachycardia and was converted to sinus rhythm with medical pharmacotherapy. Further workup with two-dimensional trans-thoracic echocardiogram and cardiac catheterization showed severe left ventricular (LV) hypertrophy in the absence of hypertension and a combined systolic and diastolic heart failure with reduced ejection fraction in the absence of significant coronary artery disease or dilated cardiac chambers. The patient denies any family or personal history of cardiac issues until the time of presentation. By exclusion, he was diagnosed with a non-ischemic cardiomyopathy secondary to his prior regimented use of anabolic steroids. Although causality can only be inferred, this case presents a potentially delayed long-term cardiac consequences of extreme AAS use over many years. Notably, our patient had remained asymptomatic, until the development of arrhythmias, eventuating in ventricular tachycardia and contributing to heart failure with reduced ejection fraction. Physicians should caution users about the risk of possible long-term cardiac complications linked with AAS use.

## Introduction

Testosterone is the main hormone responsible for male secondary sex characteristics, tissue healing and the maintenance of muscle mass. Anabolic-androgenic steroids (AAS) are synthetic derivatives of the human hormone testosterone with a more favorable anabolic-to-androgenic effect profile, metabolic breakdown pathway, and interaction with the testosterone receptor. AAS are controlled substances in the United States and have been clinically utilized for a wide range of diseases and disorders such as hypogonadism, human immunodeficiency virus, osteoporosis, short stature, anemia, severe burns, and protein-calorie malnutrition [1]. Due to their anabolic effects, AAS have been, and continue to be, misused by athletes and body-builders who seek to increase physical appearance and/or performance. However, the misuse of AAS may have unintended side effects due to the wide physiologic effects attributed to the testosterone hormone. Androgen receptors, the principal receptor acted on by testosterone and its derivatives, are located in the bone, brain, cardiovascular system, endocrine system, muscle, prostate, and hematopoietic system. Of particular interest is the effects of AAS on cardiac muscle and function. Activation of androgen receptors in the heart is thought to drive cardiac muscle growth [2]. Thus, it is probable that supra-physiologic doses of AAS, as is encountered in those that misuse AAS, may lead to left ventricular (LV) hypertrophy and/or cardiomyopathy. Recent literature supports a causal link between current AAS and cardiomyopathy [3,4]. However, the long-term cardiac sequelae in former AAS users remain to be documented. We herein present a case study of a 73-year-old prior Olympic athlete who had abused AAS for 20 years and developed a systolic and diastolic cardiomyopathy with symptomatic ventricular tachycardia secondary to severe left ventricular hypertrophy.

## Case presentation

**Figure 1:**
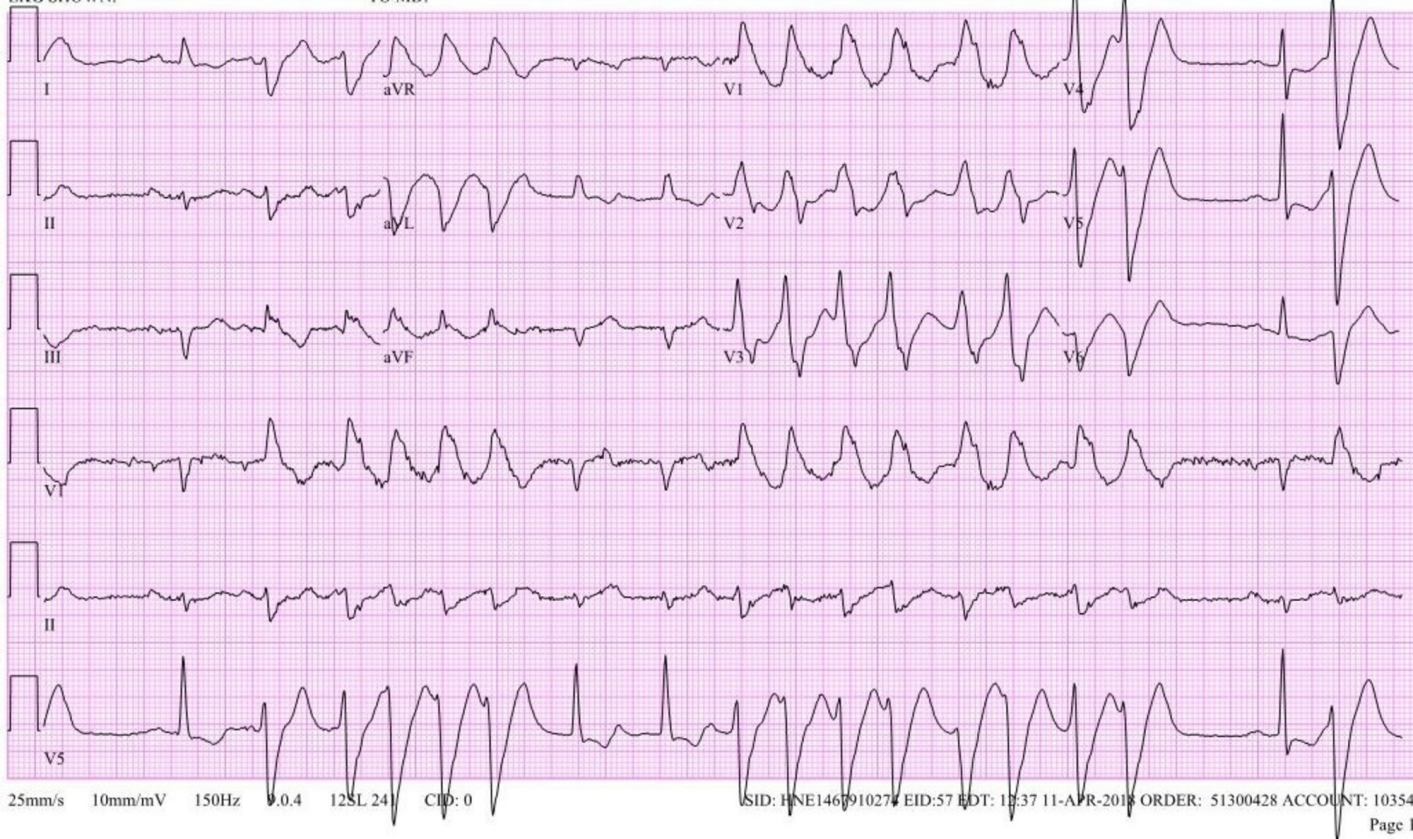
Electrocardiogram (EKG) of patient demonstrating ventricular tachycardia with occasional sinus rhythm.

**Figure 2:**
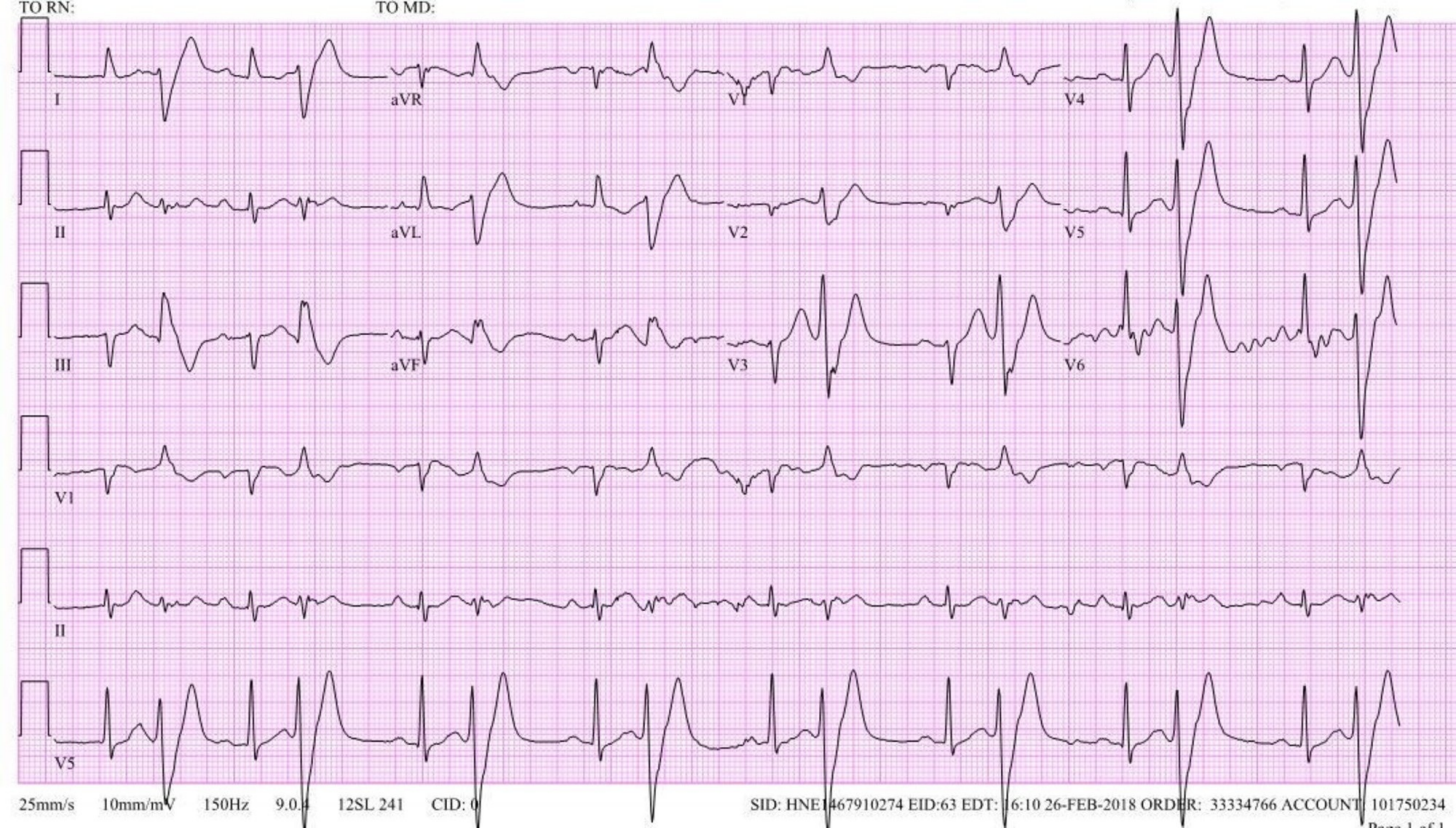
Baseline electrocardiogram (EKG) demonstrating ventricular bigeminy.

A 73-year-old man was brought to our emergency department by pre-hospital care emergency medical personnel, after he had called “911” because of progressive lightheadedness, palpitations and generalized weakness. Ventricular tachycardia was diagnosed by the pre-hospital care personnel who administered a 150 mg intravenous (IV) bolus of amiodarone. He was transported to the emergency department, where he was hemodynamically stable despite ventricular tachycardia at a rate of ~210 bpm (Figure 1). The patient’s labs were unremarkable with negative troponin. His chest X-ray showed mild pulmonary vascular congestive changes. With additional antiarrhythmic therapy, he converted to ventricular bigeminy (Figure 2). An echocardiogram showed an ejection fraction of 40–45% with regional wall motion abnormalities in a coronary distribution localized to the left ventricle. Left ventricular structural measurements suggested abnormal LV mass index and severe concentric hypertrophy. Systolic and diastolic functions were abnormal: ejection fraction and diastolic filling pressures were reduced below normal values (Table 1). He then underwent cardiac catheterization, which revealed only mild luminal irregularities in the left anterior descending and circumflex systems in addition to a 50% lesion of the mid-right coronary artery. Given the mild atherosclerotic disease noted on his coronary angiography, the overall impression was that of a non-ischemic cardiomyopathy. At this point, he underwent placement of an implantable cardioverter defibrillator (ICD). He was discharged, but discontinued amiodarone due to vague muscle aches. Subsequently, he returned two weeks later after several defibrillator discharges, in congestive heart failure, with persistent ventricular bigeminy. His worsened heart failure was ascribed to the bigeminy and responded to diuretics and control of ectopy.

**Table 1:** Left ventricular (LV) structural and function parameters. AAS: Anabolic-androgenic steroid; LVEDV: Left ventricular end diastolic volume.

LV Structural Parameters			
	Matched Normal Values	Patient	Avg. of 86 AAS users in Baggish Study (2017)
Interventricular septal thickness (mm)	11.2	15.8	12
Posterior left ventricular wall thickness (mm)	9.8	14.2	12
End-diastolic diameter (mm)	42-59	54	50
Left ventricular mass (g)	67-162	362	245
LV mass index	49-115	165 – severely abnormal	111 – within normal limits
Relative wall thickness	<0.42	0.53 – concentric hypertrophy	0.49 – concentric remodeling
Systolic Function Parameters			
Ejection Fraction (%)	55-70	35-40	52
Systolic Dimension (mm)	25-40	44.2	36
LVEDV (mL)	142	139.2	125
LVEDV index (mL/m^2^)	75 (non-dilated ventricular cavity)	64.4 (non-dilated ventricular cavity)	
Diastolic Function Parameters			
Trans-Mitral Peak E-wave (cm/s)	-	86	64
Trans-Mitral Peak A-wave (cm/s)	-	75.3	61
Trans-Mitral E-wave/A-wave Ratio	0.96 (diastolic dysfunction)	0.97 (diastolic dysfunction)	0.93 (diastolic dysfunction)

## Discussion

Systolic and diastolic heart failure had developed in this long-retired Olympic athlete, who had used AAS, including clenbuterol, in order to remain competitive with his peers. He acknowledged using AAS for approximately 20 years, having stopped usage for more than three decades. He also disclosed a seven-year history of alcohol abuse from which he had been abstinent for six years. Notably, he commented that he had experienced intermittent episodes of paroxysmal palpitations ever since he had stopped using AAS. We hypothesize that his long history of using AAS was a major contributing factor to his arrhythmia and heart failure secondary to severe LV hypertrophy. Other diagnostic considerations included ischemic and alcoholic cardiomyopathy, but catheterization demonstrated no significant coronary artery disease, non-dilated LV chambers, and he had been abstinent from alcohol for six years. Age may certainly have been another contributor to this patient’s diastolic dysfunction; however, the presence of LV hypertrophy suggests that cardiac remodeling was the stronger contributor to diastolic dysfunction (Table 1).

Additionally, hypertension did not seem to be a factor as the patient had historically never been hypertensive. This patient’s lack of concurrent systemic or extra-cardiac disease or low-voltage electrocardiogram (EKG), grossly increased left ventricular mass, presence of left ventricular hypertrophy, and the presence of a reduced ejection fraction as seen on the echocardiogram make restrictive cardiomyopathy secondary to an infiltrative pathology also unlikely [5]. End-stage or grade-four non-obstructive hypertrophic cardiomyopathy characterized by an ejection fraction less than 50% cannot be definitively ruled out without genetic testing of sarcomere proteins. However, there are other findings, or lack thereof, which is characteristic of end-stage hypertrophic cardiomyopathy not in support of this diagnosis. For example, end-stage hypertrophic cardiomyopathy is characterized by asymmetric (not concentric) hypertrophy, dilated ventricular cavity volumes approaching that of a dilated cardiomyopathy, and progressive thinning of the LV wall thickness. This patient demonstrated a concentric hypertrophy, normal LV volumes, and grossly increased relative wall thickness. Also, this type of rapid decompensation is typically seen within a span of five years in patients aged 20–40 years, not in those in their seventh decade of life. In addition, the negative family or personal history of cardiac dysfunction also does not support this diagnosis [6].

Currently, the exact mechanisms behind the pathophysiologic changes that AAS have on cardiac muscle and function are still being elucidated upon. Evidence from numerous case studies suggests that current AAS use may cause acute decompensated heart failure secondary to severe dilated cardiomyopathy that may or may not be transient in nature (Table 2) [7-18]. The dysfunction is usually severe and characterized by an ejection fraction less than 35–40%. It is unclear which factors may promote acute, systolic cardiomyopathy secondary to dilated cardiomyopathy, but contributors may include total dosage, intensity of abuse, intensity and type of training while on AAS, or genetic differences that may affect tolerance to AAS. More studies and reports may be helpful in delineating these risks factors. On the other hand, several pilot and prospective cohort studies suggest AAS causing an insidious LV hypertrophy on the heart. It is thought that the effects of AAS on inducing a phenotype of LV hypertrophy may be multifactorial, driven primarily by the presence and activation of androgen receptors on cardiac myocytes. The increased pressure and its effect on afterload burden, from the direct action of AAS and the stress encountered during training, is also thought to contribute to LV hypertrophy [2]. The effect of AAS on the heart may very well be synergistic to the type of exercise one is engaged in as well. Indeed, studies have shown that strength-trained athletes go on to develop larger increases in relative wall thickness, concentric LV hypertrophy, while exhibiting less changes in cardiac chamber dimensions even without the use of AAS. Whereas, those engaged in aerobic or endurance exercises are more likely to have larger changes in cardiac chamber dimension such as increased LV internal diameter [19]. A study by Urhausen et al. conducted a cross sectional study which showed increasing dimensions of LV wall thickness and muscle mass in current users of AAS who engaged in strength training when compared to those who were prior users and non-users. Further, these changes to the LV wall thickness and mass did not recover with discontinuation of AAS after several years [3]. More recently, a study comparing 80 weightlifters who were former or current AAS users with 80 non-AAS using weightlifters, demonstrated the development of asymptomatic cardiomyopathy in the users. Changes were observed in long-term AAS users, both current and recent, and was characterized by a persistent diastolic cardiomyopathy without dilated cardiac chambers [4]. A consistent feature in AAS users was LV diastolic dysfunction, whereas asymptomatic systolic dysfunction was a consistent finding in only current users. The data suggest that this systolic dysfunction may initially be a transient event with recovery following cessation of AAS use. It was also interesting to note that users of AAS had LV structural parameters suggestive of concentric remodeling and not LV hypertrophy at their young age (Table 1). However, it should be noted that the average age and duration of use in this cohort of AAS users was 23 and seven and a half years, respectively, which is pale in comparison to the patient presented here, who used AAS over a span of 20 years and being an elite professional athlete may very well have engaged in more vigorous strength training. Further, it is reasonable that the seemingly irreversible diastolic dysfunction associated with AAS use may progress over time by cardiac myocyte remodeling, as is often seen in other cardiomyopathies, to a phenotype such as that encountered in our patient [20]. For example, LV hypertrophy associated with hypertension eventually progresses to an end-stage heart failure characterized by a dilated cardiomyopathy.

**Table 2:** Case reports of AAS causing cardiomyopathy. AAS: Anabolic-androgenic steroid; UNK: Unknown; LVEF: Left ventricular ejection fraction; LVED: Left ventricular end diastolic.

Authors, year	Age	Cardiomyopathy	Cardiac Function Parameters	Other Associated Findings	Last Use of AAS	Duration of Use	Outcome
Schollert and Bendixen, 1993 [12]	33	Hypertrophy + dilated	UNK	Atrial flutter with two-to-one block	Three weeks prior	UNK	Did not survive
Nieminen et al., 1996 [7]	31	Hypertrophy + dilated	LVEF: 14% LVED 79 mm (severe)	Second degree Mobitz type one heart block	UNK	Several years	UNK
Ferrera et al., 1997 [8]	24	Dilated	LVEF: 39%	None reported	UNK	UNK	UNK
Vogt et al., 2002 [13]	21	Dilated	LVEF: 20-30% LVED: 80 mm (severe)	None reported	UNK	UNK	UNK
Clark and Schofield, 2005 [14]	40	Dilated	LVEF: 10-15%	Global hypokinesis	UNK	UNK	UNK
Ahlgrim and Guglin, 2009 [11]	41	Dilated	LVEF: 18% LVED: 67 mm	Global hypokinesis	Two years prior	Six weeks	Stabilized
Youssef et al., 2011 [9]	39	Dilated	LVEF: 35% LVED: 69 mm	Apical thrombus formation	Current	Three years	Stabilized LVEF: 40-45% at three-month follow-up
Shamloul et al., 2014 [15]	37	Dilated	LVEF: 13%	Multiple thrombi in ventricles and stroke	Current	Two years	Did not recover due to complications
Han et al., 2015 [16]	30	Dilated	LVEF: 15%	Atrial fibrillation w/ RVR and global hypokinesis	Six weeks prior	Seven years	Stabilized LVEF: 63% at two-year follow-up
Placci et al., 2015 [10]	25	Takotsubo (apical ballooning)	LVEF: 40%	Middle apical akinesia and compensatory hyperkinesia in basal segments	Current	Three weeks	Stabilized and complete recovery of cardiac function
Sabzi and Faraji, 2017 [17]	34	Dilated	LVEF: 30% LVED: 77 mm	Ventricular thrombus formation. Akinetic and thin septum and apex with mild mitral regurgitation	UNK	UNK	LVEF: 40-45% at three-month follow-up
Patel et al., 2018 [18]	28	Dilated	LVEF: 20%	Severe mitral stenosis, aortic regurgitation, and left atrial mass	Current	Two years	Stabilized

Our patient’s echocardiograms were consistent with observations from the aforementioned controlled studies, confirming increased left ventricular mass in a concentric manner, systolic and diastolic dysfunction, without dilated cardiac chambers best explained by a non-ischemic cardiomyopathy secondary to severe and sustained left ventricular hypertrophy from chronic anabolic steroid use (Table 1) [20]. The magnitude of the increase in left ventricular mass and concentric hypertrophy were less pronounced in the aforementioned studies; it is in line with the trend observed with AAS use. This cause of concentric hypertrophy may very well be dependent on dose, type of training, and amount of physical exertion during training.

## Conclusions

Although causality can only be inferred, this case presents a delayed long-term cardiac consequence of extreme AAS use over many years with the potential of affecting long-term morbidity and mortality. Notably, our patient had remained asymptomatic, until the development of arrhythmias, eventuating in ventricular tachycardia and contributing to heart failure with reduced ejection fraction. This deterioration in cardiac function may partly be attributed to natural complications arising from increased cardiac aging and fibrosis superimposed on a primary diagnosis of long-standing left ventricular hypertrophy from misuse of AAS. Physicians should caution users about the risk of possible long-term cardiac complications linked with AAS use with their respective exercise regimen.
